# Sex-Specific Outcomes of Transcatheter Edge-to-Edge Repair for Degenerative Mitral Regurgitation: Results From the CLASP IID Trial

**DOI:** 10.1016/j.jscai.2025.103713

**Published:** 2025-07-23

**Authors:** Molly Szerlip, Linda D. Gillam, D. Scott Lim, Jörg Hausleiter, Firas Zahr, Scott Chadderdon, Raj R. Makkar, Ralph Stephan von Bardeleben, Tobias Friedrich Ruf, Robert M. Kipperman, Andrew N. Rassi, Scott Goldman, Konstantinos Koulogiannis, Leo Marcoff, Robert L. Smith, CLASP IID Pivotal Trial Investigators

**Affiliations:** a Department of Cardiology, Baylor Scott and White: The Heart Hospital Plano, Plano, Texas; b Department of Cardiovascular Medicine, Atlantic Health System Morristown Medical Center, Morristown, New Jersey; c Division of Cardiovascular Medicine, University of Virginia Health System Hospital, Charlottesville, Virginia; d Medizinische Klinik und Poliklinik IM, Klinikum der Universität München, Munich, Germany; e Division of Cardiovascular Medicine, Oregon Health & Science University, Portland, Oregon; f Department of Interventional Cardiology, Cedars-Sinai Medical Center, Los Angeles, California; g Department of Cardiology, University Medical Centre Mainz, Mainz, Germany; h Department of Cardiology, Kaiser Permanente San Francisco Medical Center, San Francisco, California; i Department of Surgery, Lankenau Medical Center, Wynnewood, Pennsylvania

**Keywords:** MitraClip system, mitral transcatheter edge-to-edge repair, PASCAL system, sex

## Abstract

**Background:**

Female patients with degenerative mitral regurgitation (DMR) can have worse mitral valve repair outcomes. We aim to study sex-specific differences in contemporary mitral transcatheter edge-to-edge repair (M-TEER).

**Methods:**

One-year outcomes in prohibitive surgical risk patients with 3+/4+ DMR from the randomized CLASP IID trial were analyzed by sex.

**Results:**

The analysis population comprised 34.7% female patients (n = 102) and 65.3% male patients (n = 192). Female patients had significantly lower body mass index, fewer comorbidities, and smaller left ventricular indexed volumes and mitral valve area at baseline. At 1 year, there were no significant differences between female and male patients in survival (92.1% vs 90.9%, *P* = .754), freedom from heart failure hospitalization (91.7% vs 94.4%, *P* = .366), and freedom from major adverse events (86.0% vs 85.9%, *P* = .985), respectively. Both sexes achieved a significant reduction in mitral regurgitation from baseline at 1 year (*P* < .001), with comparable mitral regurgitation ≤1+ (75.6% vs 75.0%, *P* = 1.000). Baseline-adjusted changes in indexed left ventricular volumes (diastolic: –18.8 vs –15.7 mL, *P* = .087; systolic: –5.6 vs –5.0 mL, *P* = .645) and mitral gradients (+1.4 vs +1.1 mm Hg, *P* = .116) were similar, with no indication of stenosis. New York Heart Association class I/II was achieved in 86.4% of female patients vs 88.7% of male patients (*P* = .674), and the Kansas City Cardiomyopathy Questionnaire overall score increased by 14.1 vs 15.8 points (*P* = .502), respectively.

**Conclusions:**

In the CLASP IID randomized trial, both sexes experienced high survival and freedom from major adverse events and heart failure hospitalization at 1 year post–M-TEER, with comparable improvements in echocardiographic, functional, and quality-of-life measures, despite differences in clinical presentation and the procedure. Results demonstrate that female patients achieve favorable safety and effectiveness outcomes, comparable to male patients, with contemporary M-TEER in DMR.

## Introduction

Mitral regurgitation (MR) is one of the most prevalent valvular diseases worldwide, causing heart failure and significant morbidity and mortality.^1^ There are known sex-specific differences in the pathophysiology of degenerative MR (DMR)^2^ and disease progression.^3^ Prior studies suggest female patients with DMR may be underdiagnosed,^1^ underreferred,^4^ undertreated, and undergo surgical repair at a more advanced disease stage^5^ than their male counterparts. Female patients may experience worse outcomes compared to male patients^6^ with surgical repair; however, reports of mitral transcatheter edge-to-edge repair (M-TEER) demonstrate greater postprocedural reverse remodeling^7^ and improved long-term survival in female patients over male patients.^8^

We report the first sex-specific subgroup analysis of a randomized controlled trial involving M-TEER. These data come from the CLASP IID trial, which is a core lab–adjudicated, randomized controlled trial comparing 2 contemporary M-TEER therapies, the PASCAL system (Edwards Lifesciences) and the MitraClip system (Abbott).^9^^,^^10^ We aim to assess characteristics and outcomes of M-TEER between female and male patients in prohibitive surgical risk patients with severe symptomatic DMR.

## Methods

### Study design

The CLASP IID trial (NCT03706833) is a prospective, multinational, multicenter, randomized controlled trial for prohibitive surgical risk patients with significant symptomatic DMR, designed to evaluate the safety and effectiveness of M-TEER with the PASCAL system. The study details were published previously.^9^^,^^10^ Briefly, prohibitive surgical risk patients with 3+ or 4+ DMR who were deemed candidates for M-TEER with both the PASCAL system and the MitraClip system were enrolled in the trial between 2018 and 2021 and randomized to the PASCAL system or MitraClip system (2:1). The study used the original PASCAL system and the newer PASCAL Precision system (collectively referred to as the PASCAL system) with PASCAL/PASCAL Ace implants, and the MitraClip G2/G3 and G4 systems (collectively referred to as the MitraClip system) with MitraClip NT/NTR/XTR/NTW/XTW implants. Study assessments were performed at baseline, during hospital stay, at discharge (or 7 days postprocedure, whichever was earlier), with follow-up at 30 days, 6 months, 1 year, and annually through 5 years. All echocardiographic assessments were performed by an independent echocardiography core laboratory (Cardiovascular Core Lab at Atlantic Health System Morristown Medical Center) based on the American Society of Echocardiography guidelines.^11^

The study followed the Mitral Valve Academic Research Consortium guidelines, Declaration of Helsinki, Good Clinical Practice principles, and ISO 14155:2011, and approvals of the investigational review board/ethics committee at each participating center. Written informed consent was collected from all participating patients. Study oversight comprised a multidisciplinary central screening committee, an echocardiographic core laboratory, a clinical events committee, and a data safety monitoring board. The CLASP IID trial was sponsored by Edwards Lifesciences.^9^

### Statistical analysis

Baseline characteristics and safety outcomes are reported for all patients with an attempted procedure (defined as skin incision), and effectiveness outcomes are reported for all patients with an attempted device (defined as guide sheath or steerable guide inserted into the femoral vein). Continuous variables were summarized with number of observations, mean ± SD or median (Q1-Q3), and where applicable, 95% CI based on t-distribution. The *P* values were calculated using *t* test or the Kruskal-Wallis test. Categorical variables were summarized with patient percentage, count, and, where applicable, 95% exact CI. *P* values were calculated using the Fisher exact test. Left ventricular (LV) dimensions, LV and left atrial (LA) volumes, and forward stroke volumes were indexed using the baseline body surface area (BSA) according to Mosteller’s formula.^12^

Kaplan-Meier estimates were used to analyze time-to-event variables and reported with a *P* value based on the log-rank test. The exact McNemar’s test was used to assess binary repeated measures.

For continuous variables, mean changes between groups were calculated as paired change and *P* values by *t* test. Analysis of covariance (ANCOVA) model was used with baseline values and sex as covariates and report the least square means difference ± standard error and *P*_*ANCOVA*_. *P* value for interaction was calculated with baseline values, sex, treatment group, and interaction between sex and treatment group as covariates in the ANCOVA model. For categorical variables, change was calculated by paired analysis of the proportion of patients. *P* values for between-group comparison were calculated by Fisher exact test, and for interaction of the treatment group by the Breslow-Day test.

Unless noted otherwise, patients with missing data were excluded from the denominator. Statistical analyses were performed with SAS software, version 9.4 or higher (SAS Institute).

## Results

The CLASP IID trial enrolled 300 patients, comprising 34.7% (n = 104) female patients and 65.3% (n = 196) male patients. Two female patients withdrew consent and exited the study prior to the procedure. Four male patients exited prior to the procedure due to death (n = 2), withdrawal (n = 1), and randomization error by site (n = 1). The procedure was attempted in 34.7% (n = 102) of female patients and 65.3% (n = 192) of male patients. One-year follow-up was completed in 92.5% of female patients and 93.0% of male patients, with echocardiographic follow-up at 1 year in 87.1% of female patients and 87.2% of male patients.

### Baseline characteristics

Baseline characteristics are reported in Table 1. Female patients had significantly lower body mass index and smaller LV volumes after indexing by BSA (*P* < .001). Significantly fewer comorbidities were observed in female vs male patients at baseline, including dilated, ischemic or nonischemic cardiomyopathy (5.9% vs 21.9%; *P* < .001), coronary artery disease (26.5% vs 45.8%; *P* = .002), myocardial infarction (8.8% vs 19.3%; *P* = .019), coronary artery bypass graft (4.9% vs 14.1%; *P* = 0.018), percutaneous cardiovascular intervention (14.7% vs 28.6%; *P* = .009), but higher transient ischemic attack (12.7% vs 5.2%; *P* = .038) was observed. Additionally, there were significant differences between sexes in regurgitant orifice area (0.45 cm^2^ vs 0.52 cm^2^), tricuspid annular plane systolic excursion (19.9 mm vs 21.2 mm), anterior-posterior vena contracta width (5.7 mm vs 6.2 mm), and mitral valve (MV) area (5.1 mm vs 6.4 mm). The proportion of patients in New York Heart Association (NYHA) functional class III/IV was significantly higher in female patients compared to male patients at baseline (71.6% vs 58.3%), and 6-minute walk distance and Kansas City Cardiomyopathy Questionnaire (KCCQ) overall score were significantly lower (239.4 m vs 276.1 m, and 55.0 points vs 60.4 points, respectively) in female patients compared to male patients.

**Table 1:** Baseline characteristics.

Characteristics	Female patients (n = 102)	Male patients (n = 192)	*P* value
Demographics			
Age, y	81.2 ± 7.35 (102)	81.2 ± 6.93 (192)	1.000
White race	65.7% (67)	74.5% (143)	.363
Body mass index, kg/m^2^	24.4 ± 5.17 (102)	26.7 ± 5.04 (192)	<.001
STS mortality score MV repair, %	4.0 ± 2.65 (102)	3.8 ± 2.55 (192)	.459
STS mortality score MV replacement, %	6.0 ± 2.98 (102)	5.1 ± 2.93 (192)	.005
EuroScore II, %	3.9 ± 2.45 (102)	3.9 ± 3.24 (192)	.067
New York Heart Association class III/IV	71.6% (73)	58.3% (112)	.031
6-Minute walk distance, m	239.4 ± 134.46 (100)	276.1 ± 110.05 (188)	.002
KCCQ overall score	55.0 ± 22.48 (102)	60.4 ± 22.02 (191)	.048
EQ-5D-5L visual analog score	61.8 ± 19.16 (91)	62.5 ± 20.01 (171)	.596
Medical history comorbidities			
Atrial fibrillation	52.0% (53)	59.9% (115)	.216
Cardiomyopathy	5.9% (6)	21.9% (42)	<.001
Coronary artery disease (≥50% stenosis)	26.5% (27)	45.8% (88)	.002
Renal insufficiency or failure^a^	35.3% (36)	38.5% (74)	.614
Diabetes	12.7% (13)	19.3% (37)	.192
Hypertension	86.3% (88)	80.7% (155)	.260
Dyslipidemia or hyperlipidemia	64.7% (66)	69.8% (134)	.431
Myocardial infarction	8.8% (9)	19.3% (37)	.019
Peripheral arterial disease	4.9% (5)	4.7% (9)	1.000
Anemia (chronic)^c^	2.9% (3)	6.3% (12)	.275
Stroke	5.9% (6)	5.7% (11)	1.000
Transient ischemic attack	12.7% (13)	5.2% (10)	.038
Chronic obstructive pulmonary disease	17.6% (18)	15.1% (29)	.617
Pacemaker/ICD	5.9% (6)	10.9% (21)	.203
Percutaneous coronary intervention/stent	14.7% (15)	28.6% (55)	.009
Coronary artery bypass graft surgery	4.9% (5)	14.1% (27)	.018
Gastrointestinal or esophageal bleeding	8.8% (9)	6.8% (13)	.642
Home oxygen use	2.9% (3)	3.6% (7)	1.000
≥1 hospitalization in the past 12 months	36.3% (37)	30.7% (59)	.362
Prior aortic valve surgery/intervention	2.9% (3)	9.4% (18)	.055
Prior tricuspid valve surgery/intervention	1.0% (1)	0.0% (0)	.347
Echocardiographic measures			
Degenerative MR category			
Flail	72.5% (74)	78.4% (149)	.312
Prolapse	27.5% (28)	20.5% (39)	.191
Severe bileaflet prolapse	0.0% (0)	1.1% (2)	.544
MR grade^b^			.778
3+	26.5% (27)	24.5% (47)	
4+	73.5% (75)	75.5% (145)	
PISA effective regurgitant orifice area, cm^2^	0.45 ± 0.145 (65)	0.52 ± 0.215 (142)	.025
PISA regurgitant volume, mL	66.4 ± 16.94 (65)	73.2 ± 25.70 (142)	.102
Primary jet location A2-P2	88.2% (90)	85.4% (164)	.593
>1 MR jets	19.6% (20)	18.8% (36)	.877
LVEDV, mL	109.9 ± 28.52 (99)	165.1 ± 47.95 (188)	<.001
LVESV, mL	42.3 ± 15.84 (99)	71.4 ± 31.24 (188)	<.001
LVEDD, mm	53.9 ± 5.91 (100)	58.7 ± 6.20 (192)	<.001
LVESD, mm	34.7 ± 6.61 (100)	40.3 ± 7.63 (189)	<.001
LA volume, mL	114.2 ± 50.76 (102)	124.2 ± 43.64 (191)	.011
LVEDV (indexed), mL	66.4 ± 17.22 (99)	83.0 ± 22.95 (188)	<.001
LVESV (indexed), mL	25.5 ± 9.42 (99)	35.9 ± 15.18 (188)	<.001
LVEDD (indexed), mm	32.8 ± 4.72 (100)	29.6 ± 3.92 (192)	<.001
LVESD (indexed), mm	21.1 ± 4.31 (100)	20.4 ± 4.28 (189)	.264
LA volume (indexed), mL	69.6 ± 33.20 (102)	62.5 ± 21.96 (191)	.065
LV ejection fraction, %	61.9 ± 7.28 (102)	57.8 ± 8.59 (192)	<.001
LV stroke volume (indexed), mL	31.6 ± 8.59 (96)	31.1 ± 8.57 (169)	.638
Transmitral gradient	2.6 ± 1.24 (96)	2.4 ± 0.95 (179)	.404
Pulmonary artery systolic pressure, mm Hg	41.3 ± 11.80 (88)	42.2 ± 12.22 (159)	.808
TAPSE, mm	19.9 ± 4.51 (80)	21.2 ± 5.37 (151)	.042
TR grade^d^ ≥3+	96.0% (96)	97.4% (186)	.499
Mitral annular calcification (none)	41.2% (42)	43.8% (84)	.711
MV area, cm^2^	5.1 ± 1.11 (80)	6.4 ± 1.59 (150)	<.001
MV cleft in grasping area	4.3% (4)	3.3% (6)	.738
Pulmonary vein flow (S reversal)	83.6% (56)	77.8% (105)	.360
Right ventricular systolic function (normal)	86.3% (88)	79.1% (151)	.155
Flail width, mm	10.0 ± 3.09 (66)	10.4 ± 3.01 (131)	.503
Flail gap, mm	3.9 ± 1.53 (69)	4.4 ± 1.86 (140)	.114
Posterior mitral leaflet length, mm	12.1 ± 3.02 (102)	12.3 ± 3.48 (191)	.945
Vena contracta area 3D, cm^2^	0.59 ± 0.221 (36)	0.69 ± 0.309 (72)	.146
Vena contracta width AP, mm	5.7 ± 1.36 (95)	6.2 ± 1.38 (178)	.007

Values are mean ± SD (n) or % (n). *P* values based on Kruskal-Wallis test (continuous variables) or Fisher exact test (categorical variables).

3D, 3 dimensional; AP, anterior posterior; eGFR, estimated glomerular filtration rate; EQ-5D-5L, EuroQol 5-dimension 5-level; EuroSCORE, European System for Cardiac Operative Risk Evaluation; Hgb, hemoglobin; ICD, implantable cardioverter-defibrillator; KCCQ, Kansas City Cardiomyopathy Questionnaire; LA, left atrial; LV, left ventricular; LVEDD, left ventricular end-diastolic dimension; LVEDV, left ventricular end-diastolic volume; LVESD, left ventricular end-systolic dimension; LVESV, left ventricular end-diastolic volume; MR, mitral regurgitation; MV, mitral valve; PISA, proximal isovelocity surface area; STS, Society of Thoracic Surgeons; TAPSE, tricuspid annular plane systolic excursion; TR, tricuspid regurgitation.

a Defined as eGFR ≤59 mL/min/1.73 m^2^. Note that eGFR ≤25 mL/min/1.73 m^2^ was an exclusion criterion.

b MR grade assessed by transthoracic echocardiography or transesophageal echocardiography.

c Hgb ≤9 g/dL.

d Severe TR (TR 4+) was an exclusion criterion.

### Procedural outcomes

Successful implant rate was 99.0% for female patients and 98.4% for male patients (*P* = .554). There were 3 aborted procedures in male patients who did not receive an implant, either due to inability to grasp the leaflet or inability to achieve sufficient MR reduction. The procedure was aborted in 1 female patient after unsuccessful attempts at gaining transseptal access.

The mean transseptal puncture height in female patients was significantly lower than in male patients (38.4 mm vs 41.1 mm; *P* = .019). The number of devices implanted per patient was significantly lower in female patients (1.4 vs 1.6), with a higher proportion of male patients receiving 2 or 3 devices. Female patients in the PASCAL group had an average of 1.3 implants (vs 1.5 in the MitraClip group), and male patients treated in the PASCAL group had an average of 1.5 implants (vs 1.7 in the MitraClip group). Female patients had a significantly longer length of stay compared to male patients, median 2 days vs 1 day (*P* = .011) (Table 2).

**Table 2:** Procedural outcomes.

Outcomes	Female patients (n = 102)	Male patients (n = 192)	*P* value
Successful implant rate^a^	99.0% (101)	98.4% (189)	.554
Procedure time, min^b^	84.0 (61.0, 104.0)	88.0 (64.0, 125.0)	.110
Device time, min^c^	55.0 (32.0, 71.0)	59.0 (36.00, 94.0)	.128
Fluoroscopy duration, min^d^	20.0 (15.0, 32.0)	23.0 (15.0, 34.0)	.346
Transseptal puncture height, mm	38.4 ± 10.20 (64)	41.1 ± 9.32 (119)	.019
No. of devices implanted in patients who received a device	1.4 ± 0.53 (101)	1.6 ± 0.63 (189)	.009
No. of devices used, %			.023
1	64.4% (65)	49.7% (94)	.019
2	33.7% (34)	42.9% (81)	.133
3	2.0% (2)	7.4% (14)	.061
Location of implanted devices, n (%)			
A1P1	4.0% (4)	5.4% (10)	.777
A2P2	89.1% (90)	88.7% (165)	1.000
A3P3	12.9% (13)	15.6% (29)	.602
Other^d^	2.0% (2)	3.2% (6)	.717
Randomized group (PASCAL)	66.7% (68)	68.2% (131)	.795
PASCAL implant type, %			.548
PASCAL	57.4% (39)	50.0% (63)	–
PASCAL Ace	36.8% (25)	40.5% (51)	–
PASCAL and PASCAL Ace	5.9% (4)	9.5% (12)	–
Delivery system			–
PASCAL group			.462
PASCAL system	56 (82.4%)	97 (77.0%)	–
PASCAL Precision system	12 (17.6%)	29 (23.0%)	–
MitraClip group			.879
G2 or G3	27.3% (9)	31.1% (19)	–
G4	72.7% (24)	67.2% (41)	–
Mixed	0.0% (0)	1.6% (1)	–
Total length of stay for index procedure, d	2 (1, 3)	1 (1, 2)	.011

Continuous variables: mean ± SD (n) or median (Q1, Q3); *P* values based on Kruskal-Wallis test. Categorical variables: % (n); *P* values based on Fisher exact test.

a Successful implant: patients with the study device implanted, deployed as intended, and the delivery system retrieved successfully.

b Procedure time: from the procedure start (femoral vein puncture/skin incision) to the femoral vein access closure.

c Device time: from PASCAL implant system or MitraClip delivery system insertion into the left atrium to guide sheath or steerable guide removal.

d Other includes A1P2, P1P2, A2P3, P1A2, and medial commissure. Implant type and delivery system are reported for patients with PASCAL and MitraClip devices implanted and for whom implant/delivery system type was available.

### Clinical outcomes at 30 days and 1 year

**Figure 1:**
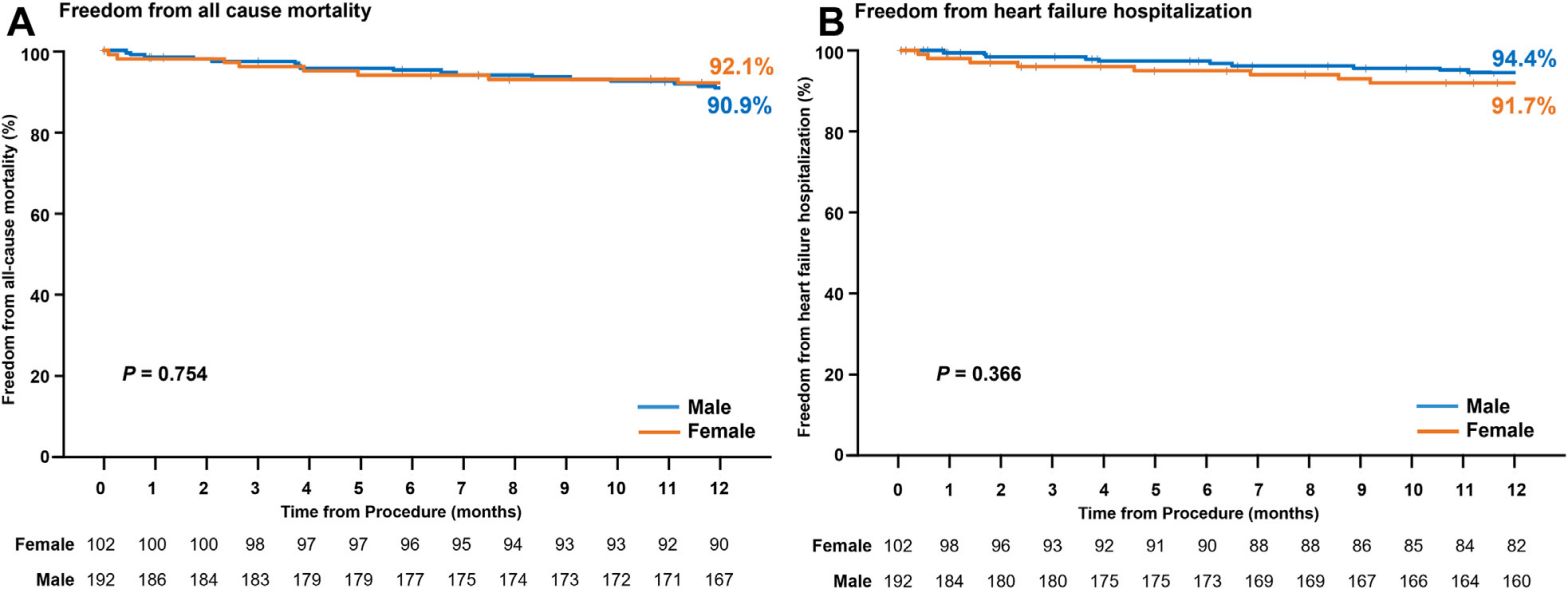
**Kaplan-Meier estimates of freedom from clinical events committee–adjudicated mortality and heart failure hospitalization.** Graphs show Kaplan-Meier analysis time to first event. Event rates at 1 year are Kaplan-Meier estimates with a *P* value from the log-rank test.

At 30 days, major adverse events (MAE) comprising cardiovascular mortality, stroke, myocardial infarction, new need for renal replacement therapy, severe bleeding and nonelective MV reintervention (either percutaneous or surgical), were reported in 14 patients (female: 5, male: 9). There was no significant difference between groups (Table 3), and no significant interaction effect of sex and treatment group on 30-day MAE (*P* > .05). Kaplan-Meier estimates for freedom from MAE at 1 year were nonsignificant, 86.0% for female patients and 85.9% for male patients (log-rank *P* = .985) (Figure 1, Table 4). There was significant interaction of sex and treatment group on the rate of freedom from MAE at 1 year (*P* = .021), with no significant difference within the PASCAL group (89.2% female patients vs 82.4% male patients; *P* = .207) and a significant difference within the MitraClip group (79.4% female patients vs 93.3% male patients; *P* = .041). (Supplemental Figure S1).

**Table 3:** CEC-adjudicated major adverse events at 30 days.

Events	Female patients (n = 102)	Male patients (n = 192)	*P* value
Cardiovascular mortality	2.0% (2)	0.5% (1)	–
Stroke	0.0% (0)	1.1% (2)	–
Myocardial infarction	0.0% (0)	0.5% (1)	–
New need for renal replacement therapy	0.0% (0)	0.0% (0)	–
Severe bleeding^a^	2.9% (3)	3.2% (6)	–
Nonelective mitral valve reintervention (either percutaneous or surgical)	2.9% (3)	0.5% (1)	–
Composite MAE rate	4.9% (5)	4.8% (9)	1.000

Values denote the cumulative number of patients with events, % (n), for 30 days. *P* value is based on Fisher's Exact test.

CEC, clinical events committee; MAE*,* major adverse events.

a Major, extensive, life-threatening, or fatal bleeding defined by the Mitral Valve Academic Research Consortium.

**Table 4:** Kaplan-Meier estimates of CEC-adjudicated events at 6 months and 1 year.

Events	Female patients (n = 102)		Male patients (n = 192)		*P* value (1 y)
	6 mo	1 y	6 mo	1 y	
Major events	8.9% (9)	14.0% (14)	8.5% (16)	14.1% (26)	.985
Cardiovascular mortality	3.9% (4)	5.0% (5)	2.2% (4)	5.0% (9)	.957
Stroke	0.0% (0)	2.1% (2)	1.0% (2)	1.6% (3)	.811
Myocardial infarction	0.0% (0)	0.0% (0)	0.5% (1)	0.5% (1)	.463
New need for renal replacement therapy	0.0% (0)	1.1% (1)	1.1% (2)	1.1% (2)	.954
Severe bleeding^a^	5.0% (5)	9.3% (9)	4.8% (9)	8.3% (15)	.770
Nonelective mitral valve reintervention (either percutaneous or surgical)	3.0% (3)	3.0% (3)	1.6% (3)	1.6% (3)	.433
Other events					
All-cause mortality	5.9% (6)	7.9% (8)	4.8% (9)	9.1% (17)	.754
Heart failure hospitalization	5.1% (5)	8.3% (8)	2.7% (5)	5.6% (10)	.366
Transient ischemic attack	1.0% (1)	1.0% (1)	0 (0.0%)	0.6% (1)	.649
Major vascular events	–	0.0% (0)	–	0.0% (0)	–
New onset of permanent atrial fibrillation	–	0.0% (0)	–	0.0% (0)	–
GI complications requiring surgery	–	0.0% (0)	–	0.0% (0)	–

Values denote Kaplan-Meier estimate, % (events), for 6 months and 1 year. *P* value is calculated from the log-rank test comparing outcomes by sex at 1 year. All enrolled patients were followed for at least 30 days or had a major adverse event.

CEC, clinical events committee.

a Major, extensive, life-threatening, or fatal bleeding defined by the Mitral Valve Academic Research Consortium.

Kaplan-Meier estimates were comparable between female and male patients for survival (*P* =0.754) (Figure 1), freedom from cardiovascular mortality (*P* = .957) (Table 4), freedom from heart failure hospitalization (HFH) (*P* = .366) (Figure 1), and freedom from all-cause mortality or HFH (*P* = .585) (Central Illustration). There was no significant interaction effect of treatment group and sex on the rate of freedom from all-cause mortality or HFH at 1 year (*P* = .389), with 84.9% each for female and male patients in the PASCAL group, and 85.3% vs 91.7%, respectively in the MitraClip group (Supplemental Figure S1).

**Central Illustration:**
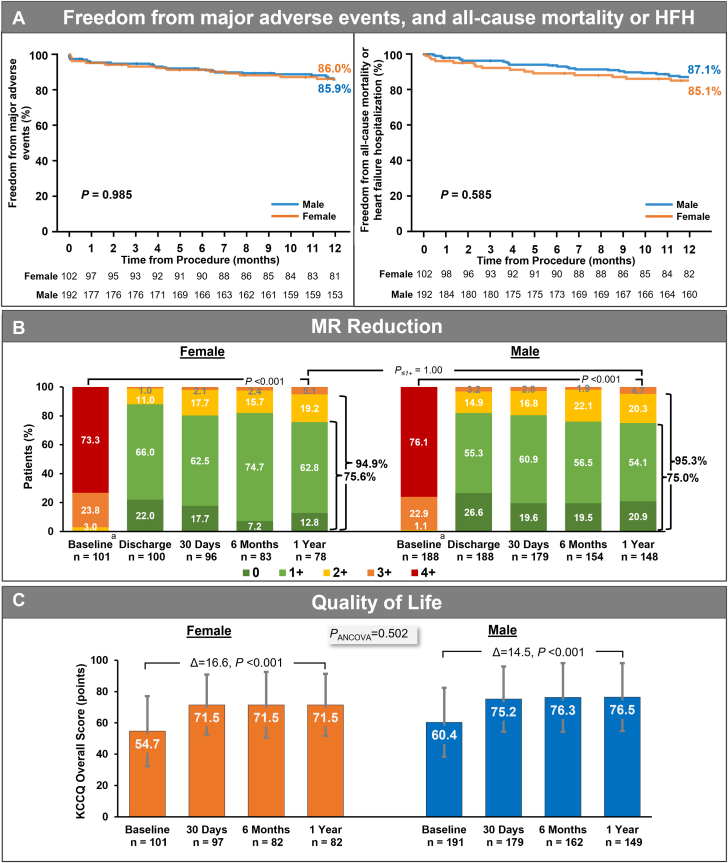
**Sex-specificoutcomes of degenerative mitral regurgitation (MR)—the CLASP IID trial.** (A) Kaplan-Meier estimates for freedom from major adverse events and freedom from all-cause mortality or heart failure hospitalization (HFH). *P* values calculated from log-rank test. (B) MR severity by transthoracic echocardiography assessed by an echocardiographic core laboratory (Atlantic Health System Morristown Medical Center). ^a^Transesophageal echocardiography was used for baseline qualification of 5 patients. The graph shows unpaired analysis. *P* values relative to baseline were calculated from paired analysis using the Wilcoxon signed-rank test, and *P* values between discharge and 1 year for MR ≤1+ were calculated using the exact McNemar’s test. (C) The graph shows unpaired analysis (mean ± SD). *P* values for intragroup comparisons were calculated from paired analysis using *t* test, and *P* values for intergroup comparisons were calculated from analysis of covariance (ANCOVA) model adjusted for baseline values as covariates. KCCQ, Kansas City Cardiomyopathy Questionnaire.

### Echocardiographic outcomes

Both sexes experienced significant MR reduction from baseline to 1 year (*P* < .001) (Central Illustration). At discharge, MR ≤2+ was achieved in 99.0% of female patients and 96.8% of male patients, and MR ≤1+ in 88.0% of female patients and 81.9% of male patients. At 1 year, the proportions of female and male patients achieving MR ≤2+ were 94.9% and 95.3%, and those achieving MR ≤1+ were 75.6% and 75.0%, respectively. The outcomes were comparable between sexes (*P* = 1.000).

**Figure 2:**
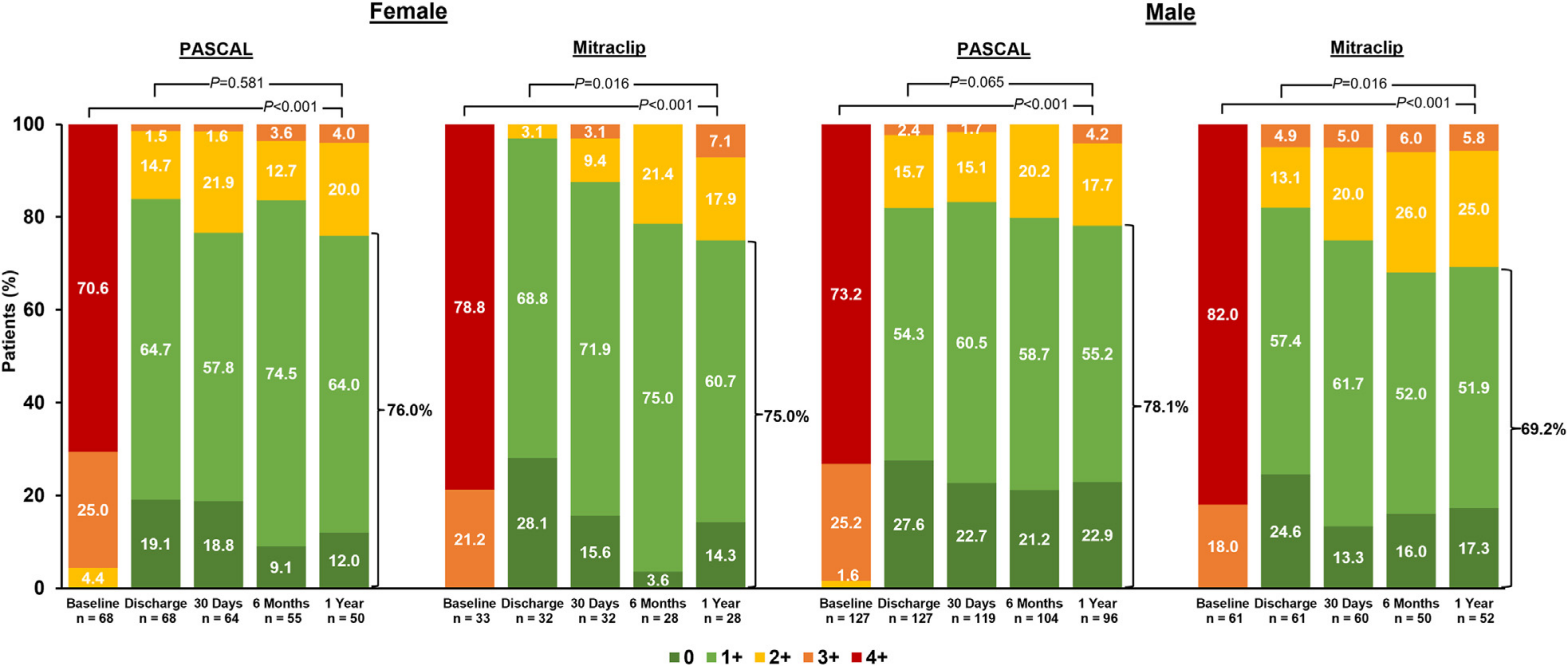
**Mitral regurgitation (MR) severity from transthoracic echocardiography by echocardiographic core laboratory, stratified by sex and treatment group.** The echocardiographic core laboratory was at the Atlantic Health System Morristown Medical Center. Transesophageal echocardiography was used for baseline qualification of 5 patients. The graph shows unpaired analysis. *P* values relative to baseline were calculated from paired analysis using the Wilcoxon signed-rank test, and *P* values for change in proportion of patients with MR ≤1+ from discharge to 1 year were calculated from paired analysis by McNemar’s test.

By treatment group, the proportion of patients with MR ≤1+ in female and male patients was 76.0% vs 78.1% when treated with the PASCAL system, compared to 75.0% vs 69.2% when treated with the MitraClip system, respectively. In the PASCAL group, the proportion of MR ≤1+ was sustained from discharge to 1 year in both female and male patients (*P* = .581 and *P* = .065, respectively), whereas it declined significantly in both groups receiving the MitraClip system (*P* = .016 and *P* = .016, respectively) (Figure 2).

**Figure 3:**
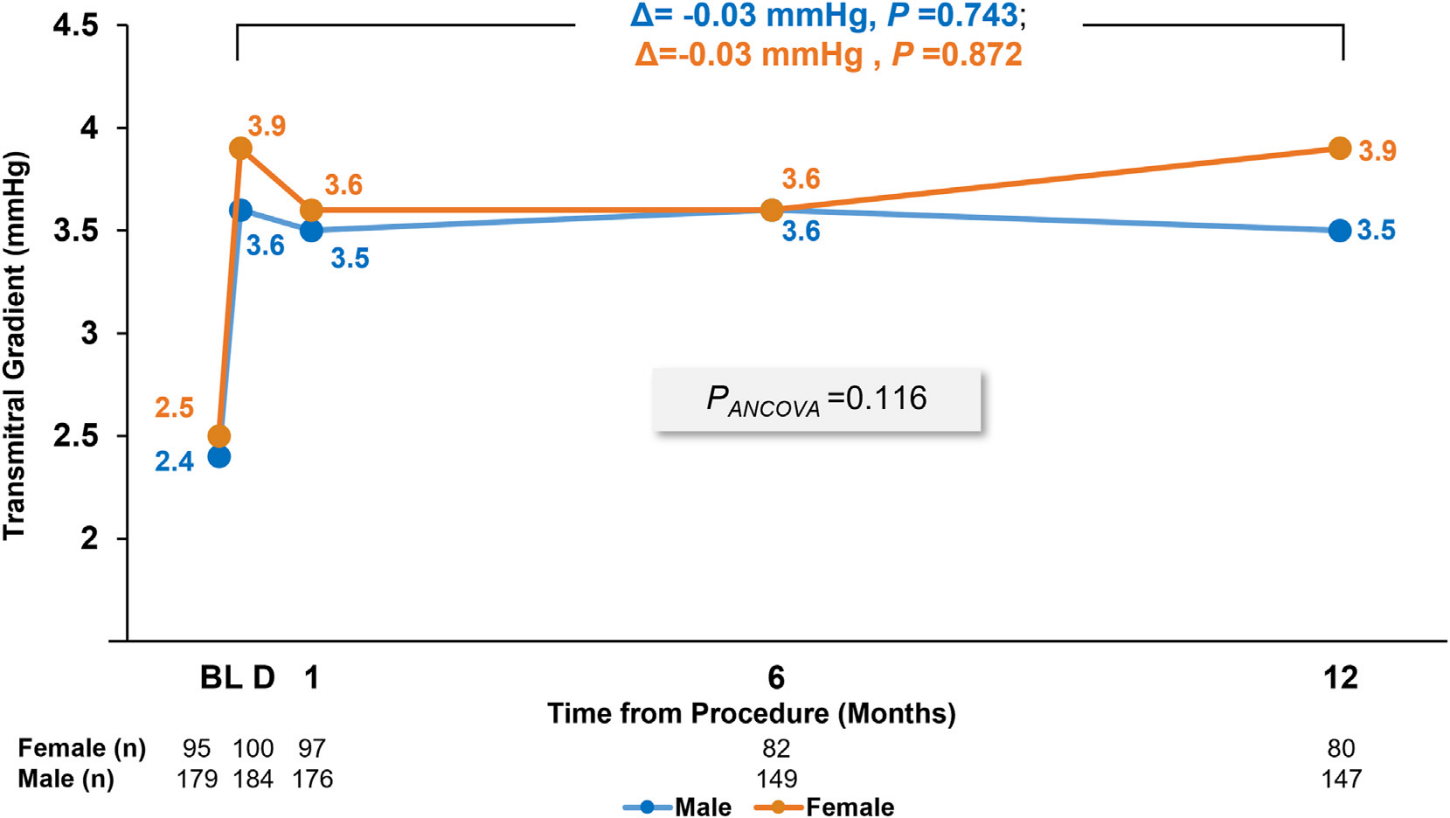
**Transmitral gradients at 1 year assessed by an echocardiographic core laboratory.** The graph shows mean values. Δ represents change from baseline (BL) to 1 year from paired analysis. Intragroup *P* values were calculated using *t* test. Analysis of covariance (ANCOVA) model was used for calculating *P*_*ANCOVA*_ with sex and BL values as covariates. D, discharge.

Postprocedure gradients were stable with a comparable and nonsignificant paired change from discharge to 1 year of –0.03 mm Hg (*P* = .872) for female patients and –0.03 mm Hg (*P* = .743) for male patients (Figure 3). There was no significant difference in outcomes between sexes (*P* = .116) (Table 5). At 1 year, mean gradients in female and male patients treated with the PASCAL system were 4.0 and 3.7 mm Hg, and with the MitraClip system were 3.8 and 3.2 mm Hg, respectively (Supplemental Figure S2). No significant interaction effect of treatment group and sex was observed. No patients were identified with hemodynamic parameters indicative of mitral stenosis per ACC/AHA guidelines (MV area ≤1.5 cm^2^ with pulmonary artery systolic pressure [PASP] >50 mm Hg and an increase in LA volume).^13^ Atrial septal defect closure was performed in 4 patients (2 female patients with 1 M-TEER implant each, and 2 male patients with 2 and 3 M-TEER implants, respectively). Echocardiographic assessment reported the presence of an atrial septal defect in 83.8% of male patients and 86.7% of female patients at discharge, 70.5% and 71.2% at 30 days, 57.1% and 67.3% at 6 months, and 52.4% and 60.5% at 1 year, respectively.

**Table 5:** Changes in echocardiographic parameters from baseline to 1 year.

	Female patients				Male patients				LSM difference	*P*_*ANCOVA*_
	Baseline	1 year	Change	*P*	Baseline	1 year	Change	*P*		
LVEDV	108.6	84.5	–24.0 ± 19.63	<.001	163.4	127.7	–35.7 ± 27.73	<.001	7.3 (–0.12, 14.71)	.054
LVEDV_indexed_	65.2	50.5	–14.7 ± 12.48	<.001	82.8	64.8	–18.0 ± 14.29	<.001	3.1 (–0.46, 6.64)	.087
LVESV	41.9	35.7	–6.2 ± 10.25	<.001	69.9	57.8	–12.1 ± 18.63	<.001	2.1 (–2.58, 6.69)	.383
LVESV_indexed_	25.1	21.3	–3.7 ± 6.22	<.001	35.4	29.4	–6.0 ± 9.94	<.001	0.6 (–1.87, 3.01)	.645
LVEF	61.7	58.6	–3.0 ± 6.62	<.001	58.1	55.9	–2.3 ± 6.26	<.001	–0.5 (–2.07, 1.10)	.546
PASP	41.4	38.9	–2.5 ± 10.74	.069	42.4	37.3	–5.1 ± 11.58	<.001	–2.1 (–4.85, 0.72)	.144
Pulmonary vein systolic flow reversal^a^	83.3%	1.9%	80%	–	77.8%	9.3%	65.4%	–	–	.101^a^
LA volume	111.7	90.6	–21.1 ± 20.94	<.001	119.4	104.2	–15.2 ± 25.29	<.001	8.2 (2.21, 14.11)	.007
LA volume_indexed_	67.6	54.7	–12.9 ± 13.41	<.001	60.3	52.6	–7.6 ± 12.69	<.001	3.2 (0.03, 6.45)	.048
Transmitral gradient	2.5	3.9	1.4 ± 1.83	<.001	2.4	3.5	1.1 ± 1.33	<.001	–0.3 (–0.75, 0.08)	.116
LV stroke volume	52.3	55.2	2.9 ± 11.79	.054	62.4	69.1	6.7 ± 16.73	<.001	9.0 (4.93, 12.98)	<.001
LV stroke volume_indexed_	31.8	33.4	1.6 ± 7.26	.075	31.7	35.0	3.4 ± 8.09	<.001	1.7 (–0.28, 3.64)	.093

Values are for paired analysis from baseline to 1 year and the *P* value is calculated by *t* test. LSM difference between groups and *P*_*ANCOVA*_ are calculated using the ANCOVA model with baseline values and sex as covariates.

ANCOVA, analysis of covariance; LA, left atrial; LSM, least square mean; LV, left ventricular; LVEDV, left ventricular end-diastolic volume; LVESV, left ventricular end-diastolic volume; PASP, pulmonary artery systolic pressure.

a Change is calculated by the mean change in proportion of patients with flow reversal. *P* values for between-group comparison of proportion of patients with flow reversal at 1 year (male vs female patients) calculated from Fisher exact test.

**Figure 4:**
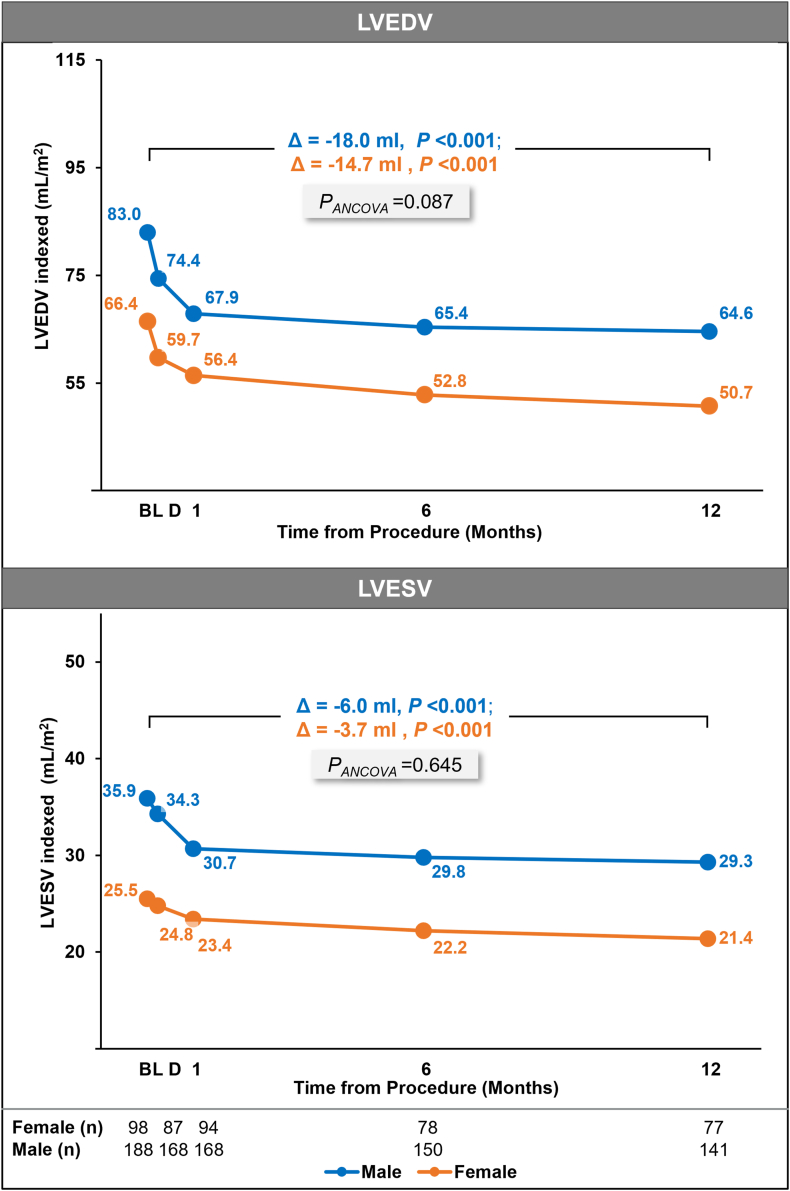
**Indexed left ventricular end-diastolic and end-systolic volumes at 1 year assessed by an echocardiographic core laboratory.** Graphs show mean values. Δ represents change from baseline (BL) to 1 year from paired analysis. Intragroup *P* values were calculated using *t* test. Analysis of covariance (ANCOVA) model was used for calculating *P*_*ANCOVA*_ with sex and BL values as covariates. D, discharge; LVEDV, left ventricular end-diastolic volume; LVESV, left ventricular end-systolic volume.

Left ventricular end-diastolic volumes demonstrated a significant change of –24.0 mL from baseline to 1 year for female patients (*P* < .001) and –35.7 mL for male patients (*P* < .001) (Figure 4, Table 5). Between-group comparisons adjusted by baseline values demonstrated a least square mean difference of 7.3 mL between sexes (*P* = .054), which reduced to 3.1 mL when indexed to baseline BSA (*P* = .087). LV end-systolic volumes significantly changed by –6.2 mL (*P* < .001) in female patients and –12.1 mL (*P* < .001) in male patients. There was no significant difference between groups, with the intergroup least square mean difference of 2.1 mL (*P* = .383), which reduced to 0.6 mL (*P* = .645) when indexed. No significant interaction of treatment group and sex was observed at 1 year (Supplemental Figure S3).

Pulmonary vein flow and LV ejection fraction similarly demonstrated significant change from baseline for both sexes, with comparable outcomes for female and male patients (Table 5, Supplemental Figure S4).

Improvement from baseline to 1 year in PASP and LV stroke volume was not significant in female patients (–2.5 mm Hg; *P* = .069 and 2.9 mL; *P* = .054, respectively), but significant in male patients (–5.1 mm Hg; *P* < .001 and 6.7 mL; *P* < .001, respectively). Between-group difference was nonsignificant for PASP (–2.1 mm Hg; *P* = .144), and significant for LV stroke volume (9.0 mL; *P* < .001), which diminished on indexing (1.7 mL; *P* = .093).

Left atrial volume demonstrated significant improvement from baseline to 1 year for both female and male patients. Between-sex differences were significant (8.2 mL; *P* = .007), and the significance persisted with indexing (3.2 mL; *P* = .048) (Table 5, Supplemental Figure S4).

### Functional and quality-of-life outcomes

**Figure 5:**
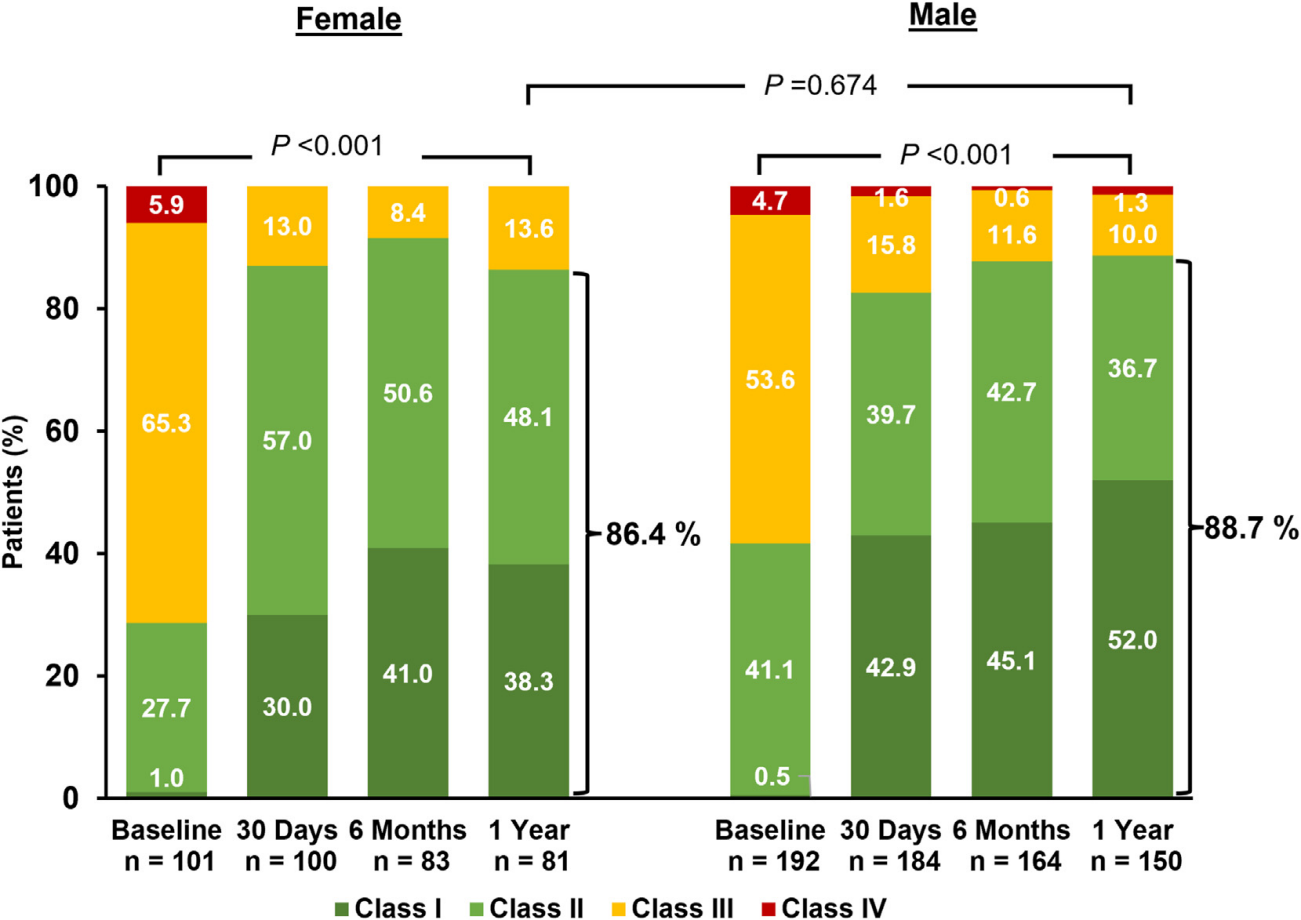
**New York Heart Association (NYHA) functional class at 1 year.** The graph shows unpaired analysis for NYHA functional class. *P* values for intragroup comparison of baseline to 1 year were calculated from paired analysis using the Wilcoxon signed-rank test. *P* values for intergroup comparison of NYHA class I/II at 1 year were calculated using Fisher exact test.

Significant improvement was observed in NYHA functional class for both sexes (*P* < .001), with 86.4% of female patients and 88.7% of male patients at NYHA class I/II at 1 year (Figure 5). Both female and male patients demonstrated significant improvements in KCCQ overall score (+16.6 points and +14.5 points) and EuroQol 5 dimension visual analog score visual analog score (+8.2 points and +9.2 points), respectively (all *P* < .05) (Central Illustration, Supplemental Figure S5). Six-minute walk distance improved in both groups, but the improvement was only significant in male patients (female patients: +15.1 m from baseline, *P* = .332; male patients: +20.1 m from baseline, *P* = .025) (Supplemental Figure S5). One-year outcomes were comparable for both sexes, and no significant difference was observed between groups when adjusted by baseline values for KCCQ overall score (*P* = .502), EuroQol 5 dimension visual analog score (*P* = .454), and 6-minute walk distance (*P* = .116). There was no significant interaction of the treatment group and sex for these outcomes (Supplemental Figures S6 and S7).

## Discussion

This sex-specific subgroup analysis of the CLASP IID randomized trial demonstrated similar safety and efficacy outcomes in female patients, comparable to those in male patients. As in most randomized trials, female patients comprised a minority of the study population (34.7%, n = 102). Even though female patients had fewer comorbidities than their male counterparts, their Society of Thoracic Surgeons predicted risk of mortality scores for MV replacement and repair were higher. This may be attributed to the significantly lower mean body mass index and the smaller LV dimensions and volumes in female patients. Consistent with known sex-specific anatomical differences in the presentation of DMR, female patients had a significantly higher proportion of MV prolapse and lower indexed LV volumes.^3^ Both MR severity and the extent of right heart disease/hemodynamics, including regurgitation volume, PASP, tricuspid annular plane systolic excursion, and tricuspid regurgitation grade, were comparable between the sexes. The fewer comorbidities in female patients, similar age, and less advanced disease stage with comparable right heart function and hemodynamic parameters for both sexes likely reflect the accumulating physician experience with M-TEER and increasing awareness and patient access to M-TEER. However, female patients were still undertreated, comprising only a third of the patient population, and were more symptomatic at baseline with higher NYHA classification scores, shorter 6-minute walk distances, and lower KCCQ scores, indicating a worse functional and quality-of-life status at baseline than male patients. Even though women were frail with more symptoms at baseline, they achieved similar 30-day and 1-year outcomes compared to men in survival, freedom from HFH, and freedom from MAE. The significantly higher MAE in women compared to men within the MitraClip group has been reported in prior studies,^14^ and given the small number of patients in the MitraClip group in this analysis, a hypothesis is difficult to formulate. It is reassuring that overall rates of MAE with contemporary M-TEER are comparable between female and male patients.

Stroke, bleeding, reinterventions, and HFH rates were observed to be numerically higher in female patients, as noted in prior M-TEER studies,^15^, ^16^, ^17^ but none were significantly different from the rates reported in male patients. Female patients had significantly lower transseptal puncture height and fewer devices implanted compared to male patients, which may be attributed to the smaller valve areas in female patients. It is noteworthy that there was no significant difference in transmitral gradients postprocedure or in follow-up between male and female patients, and no occurrence of mitral stenosis. Female patients did, however, have a longer length of stay than male patients. This may be due to the worse functional status with higher NYHA classification in female patients at baseline. A possible reason for this heightened symptomatology in female patients could be the presence of diastolic dysfunction and heart failure with preserved ejection fraction, which may have resulted in female patients needing an extra day of diuresis. Social causes may be another possible explanation for the additional length of stay, as female patients are often primary caretakers and may not have their own caretaker post-hospitalization. These observations emphasize the importance of optimizing female patients prior to the M-TEER procedure.

Both female and male patients had a similar significant reduction in MR, with a high proportion achieving MR ≤1+. Similar to prior reports, MR ≤1+ outcomes were sustained to 1 year in both sexes when treated with the PASCAL system.^9^^,^^10^ It is worth investigating if this trend is maintained over a longer follow-up. There were significant reductions in LV end-diastolic volumes and LV end-systolic volumes, with and without indexing by BSA, bringing them closer to the age and sex adjusted mean values in a normal/healthy population^18^ and indicating favorable LV remodeling in both female and male patients. Between-sex disparities were only seen via a significantly larger reduction in LA volume in female patients, and a smaller increase in LV forward stroke volume at 1 year, in accordance with known sex-specific differences in the normal range.^19^^,^^20^ Similar trends were observed in both PASCAL and MitraClip groups.

Overall, despite being frailer and more symptomatic at baseline, female patients had similar improvement in NYHA classification and quality of life by KCCQ as male patients. Although most M-TEER trials have shown similar outcomes between the sexes, some have not. The Transcatheter Mitral Valve Interventions registry demonstrated lower NYHA functional class improvement in female patients at 1 year,^21^ and the COAPT trial indicated that female patients may have a diminished benefit in reducing HFH with M-TEER compared to male patients.^15^ A 2014 analysis of functional MR patients from the GRASP registry reported similar findings despite the female patients presenting with a worse clinical profile at baseline. Their outcomes, including functional status and echocardiographic outcomes, were similar to their male counterparts’ post–M-TEER.^22^ The European Registry of Transcatheter Repair for Secondary Mitral Regurgitation study also showed no differences in quality-of-life and symptomatic improvements between sexes at 1 year.^23^ Additionally, 2 recent meta-analyses of patients undergoing M-TEER demonstrated that although there were significant differences in the baseline characteristics and procedural outcomes for female patients as compared to male patients, these differences did not affect short- or long-term outcomes.^16^^,^^24^ These findings align with the observations in this subgroup analysis from the CLASP IID trial, wherein female patients presented with worse functional status, yet achieved similar 30-day and 1-year outcomes following M-TEER.

These results show that noncomplex, M-TEER suitable patients achieve significant favorable changes with M-TEER regardless of sex. The lower proportion of female patients enrolled in this contemporary study corroborates the continued underreferral and undertreatment of female patients for mitral repair and emphasizes the need for equitable referral and practice patterns for female patients with DMR as compared to male patients. A higher index of suspicion and lower threshold for referral of female patients with DMR for M-TEER appears warranted to reduce treatment delays and minimize disease severity at the time of intervention. Utilizing KCCQ, an objective assessment for quality of life at presentation, can help in the timely identification of potential M-TEER candidates. The comparable outcomes between female and male patients, despite significant differences in baseline characteristics and symptomatology, suggest that M-TEER is a safe and effective therapy for female patients with DMR at prohibitive surgical risk. These results highlight the importance of shared decision-making and a personalized approach to the diagnosis and treatment of valvular heart disease.

### Limitations

The CLASP IID trial was not powered to assess differences between sexes. Treatment allocation was unblinded and could have introduced bias in outcome assessment. Rigorous oversight by independent adjudication committees for clinical events and echocardiographic outcomes aimed to mitigate this risk. In the trial, multiple iterations of the implants and delivery systems were used. Imbalance in device usage due to 2:1 randomization could be a potential confounder in observed results. The introduction of different device generations at varying time points and a limited sample size hinders meaningful analysis of the influence of individual device iterations and delivery systems on sex-specific outcomes.

## Conclusion

One-year results from the CLASP IID trial stratified by sex demonstrate that M-TEER is safe for both female and male patients with significant symptomatic DMR (3+ or 4+) who are at prohibitive risk for MV surgery. Despite differences in baseline characteristics, procedural outcomes, and other echocardiographic parameters, male and female patients experienced favorable clinical and echocardiographic outcomes, with comparable and significant improvements in functional and quality-of-life outcomes with contemporary M-TEER.
