# Supplementary material for: Sex-Specific Outcomes of Transcatheter Edge-to-Edge Repair for Degenerative Mitral Regurgitation: Results From the CLASP IID Trial
Source: J Soc Cardiovasc Angiogr Interv. 2025 Jul 23;4(8):103713. doi: 10.1016/j.jscai.2025.103713 (PMC12462154; doi:10.1016/j.jscai.2025.103713)
Supplement: Supplementary Figures [file mmc1.docx]

**Supplementary Appendix**

[**Supplemental Figure S1: Freedom from CEC adjudicated A) major adverse events and B) all-cause mortality or HFH, stratified by sex and treatment group.** 2](#_Toc199920882)

[**Supplemental Figure S2: Transmitral gradients, stratified by sex and treatment group as assessed by echocardiographic core laboratory.** 3](#_Toc199920883)

[**Supplemental Figure S3: Changes in left ventricular end diastolic and systolic volumes, stratified by sex and treatment group as assessed by echocardiographic core laboratory.** 4](#_Toc199920884)

[**Supplemental Figure S4: Post-procedure hemodynamic improvements: A) PASP, B) Pulmonary vein flow, C) LA Volume, and D) LV Forward stroke volume to 1 year.** 5](#_Toc199920885)

[**Supplemental Figure S5. Six-minute walk distance and EQ5D5L visual analog score at 1 year.** 6](#_Toc199920886)

[**Supplemental Figure S6: NYHA Functional Class at 1 year stratified by sex and treatment group.** 7](#_Toc199920887)

[**Supplemental Figure S7: KCCQ overall score at 1 year stratified by sex and treatment group.** 8](#_Toc199920888)

# **Supplemental Figure S1: Freedom from CEC adjudicated A) major adverse events and B) all-cause mortality or HFH, stratified by sex and treatment group.**


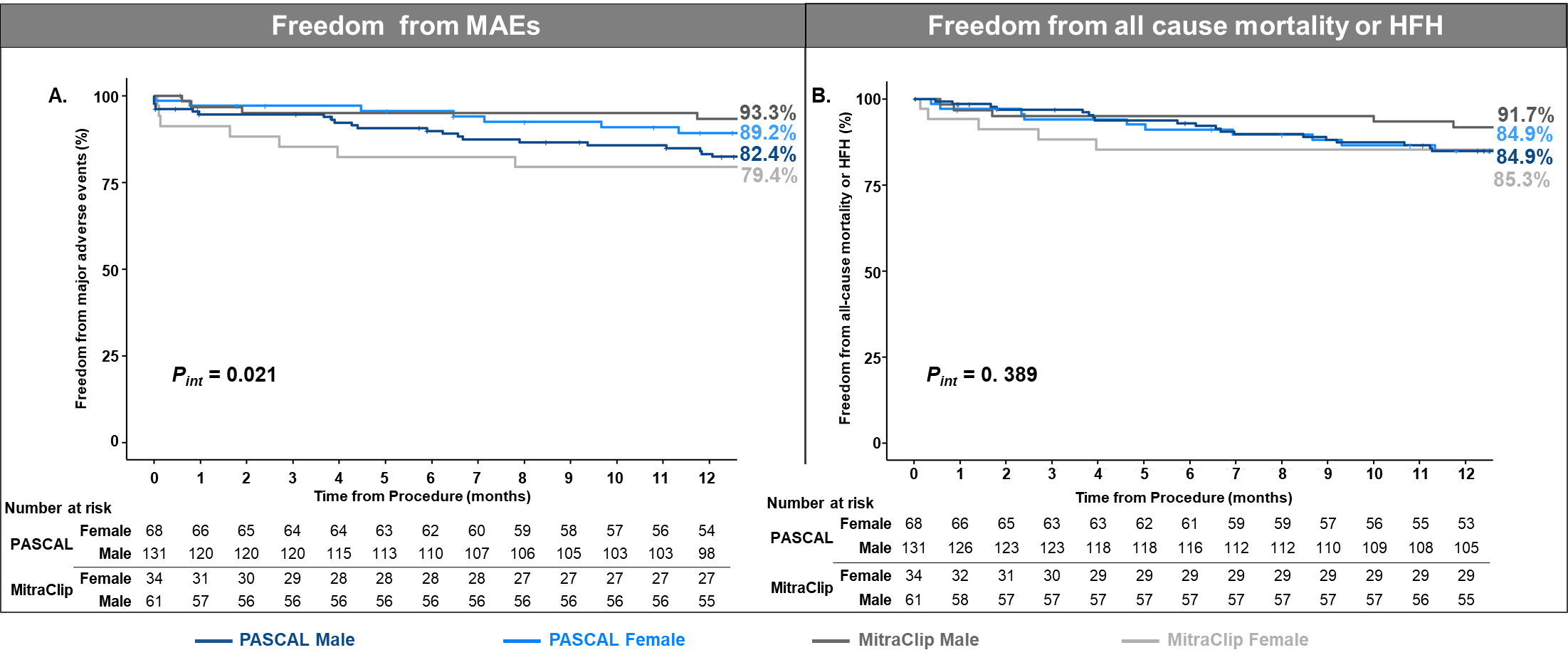


The graph shows Kaplan-Meier estimates, with interaction *P* value from cox proportional hazards model with sex and treatment device and their interaction as covariates. *CEC,* Clinical Events Committee; *MAE,* Major adverse events; *HFH,* Heart failure hospitalization.

# **Supplemental Figure S2: Transmitral gradients, stratified by sex and treatment group as assessed by echocardiographic core laboratory.**


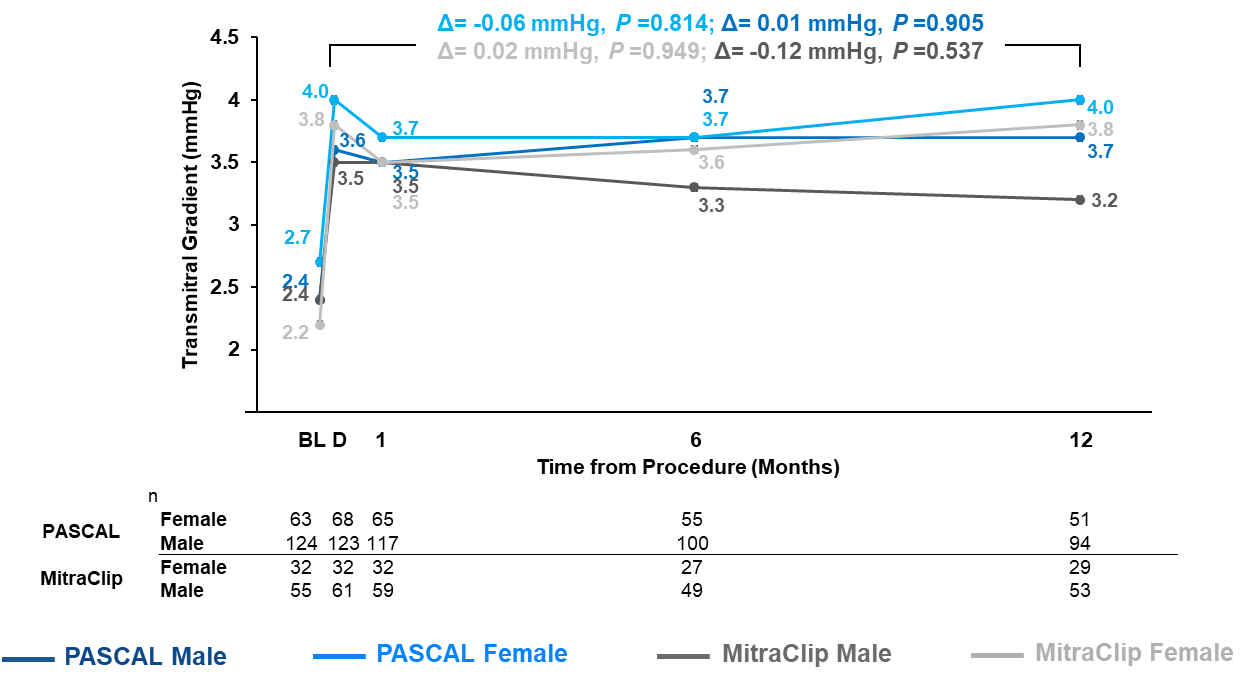


Graphs show mean values. Δ denotes paired change from discharge to 1 year, with intragroup *P* values calculated from Student’s t-test. *BL,* Baseline; *D,* Discharge.

# **Supplemental Figure S3: Changes in left ventricular end diastolic and systolic volumes, stratified by sex and treatment group as assessed by echocardiographic core laboratory.**


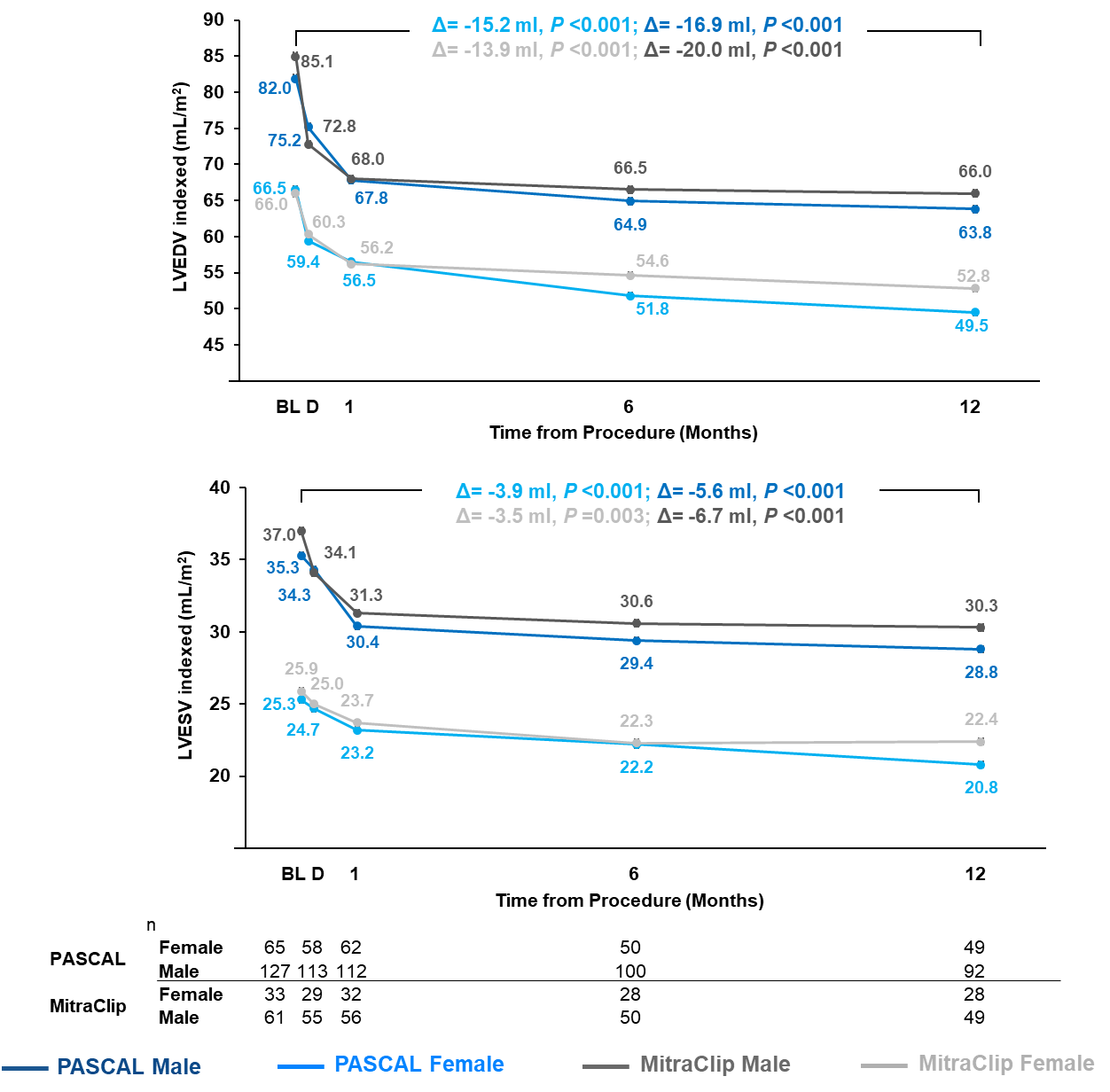


Graphs show mean values. Indexing is done by Mosteller’s formula. Δ denotes paired change from baseline to 1 year, with intragroup *P* values calculated from Student’s t-test. *BL,* Baseline; *D,* Discharge; *LVESV*, Left ventricular end-systolic volume; *LVEDV*, Left ventricular end-diastolic volume.

# **Supplemental Figure S4: Post-procedure hemodynamic improvements: A) PASP, B) Pulmonary vein flow, C) LA Volume, and D) LV Forward stroke volume to 1 year.**


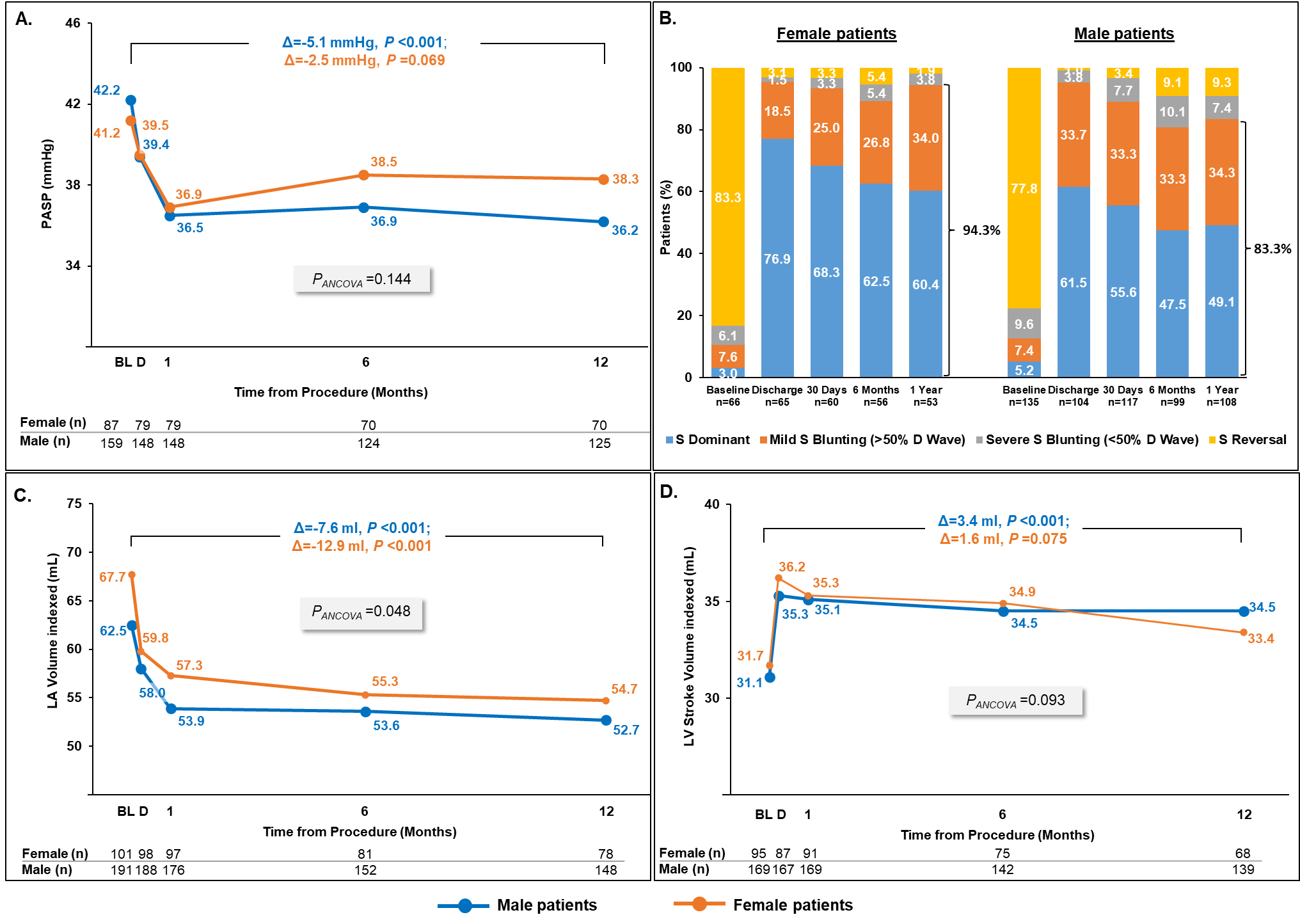


A), C) and D) Data shows mean values. Indexing is done by Mosteller’s formula. Δ denotes paired change from baseline to 1 year, with intragroup *P* values calculated from Student’s t-test. Intergroup *P* values are calculated from analysis-of-covariance (ANCOVA) model adjusted by baseline values. *BL*, Baseline; *D,* Discharge; *PASP,* Pulmonary artery systolic pressure; *LA,* Left atrial; *LV,* Left ventricular.

# **Supplemental Figure S5. A) Six-minute walk distance and B) EQ5D5L visual analog score to 1 year.**


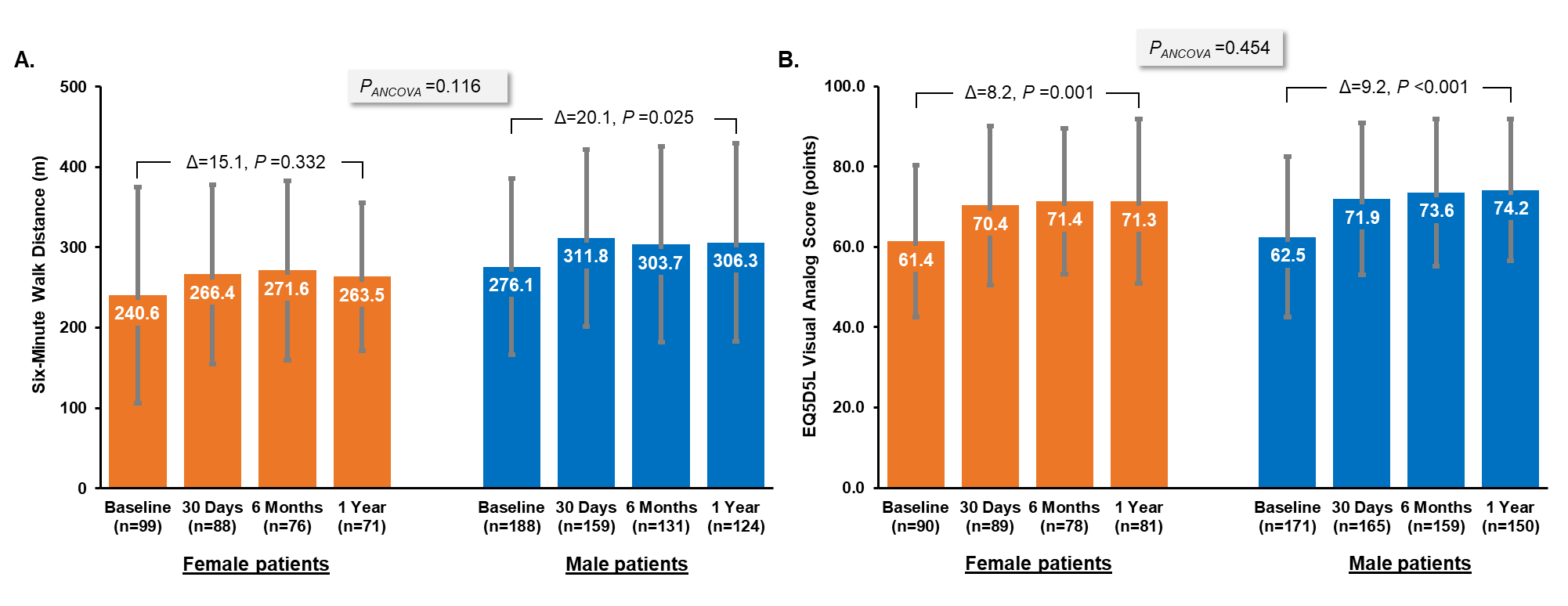
Graphs show unpaired analysis (mean ± SD). Δ denotes paired change from baseline to 1 year, with intragroup *P* values calculated from Student’s t-test. *P* values for intergroup comparisons were calculated with analysis of covariance (ANCOVA) model adjusted by baseline values. *EQ5D5L,* EuroQol 5 Dimension 5 Level.

# **Supplemental Figure S6: NYHA Functional Class at 1 year stratified by sex and treatment group.**


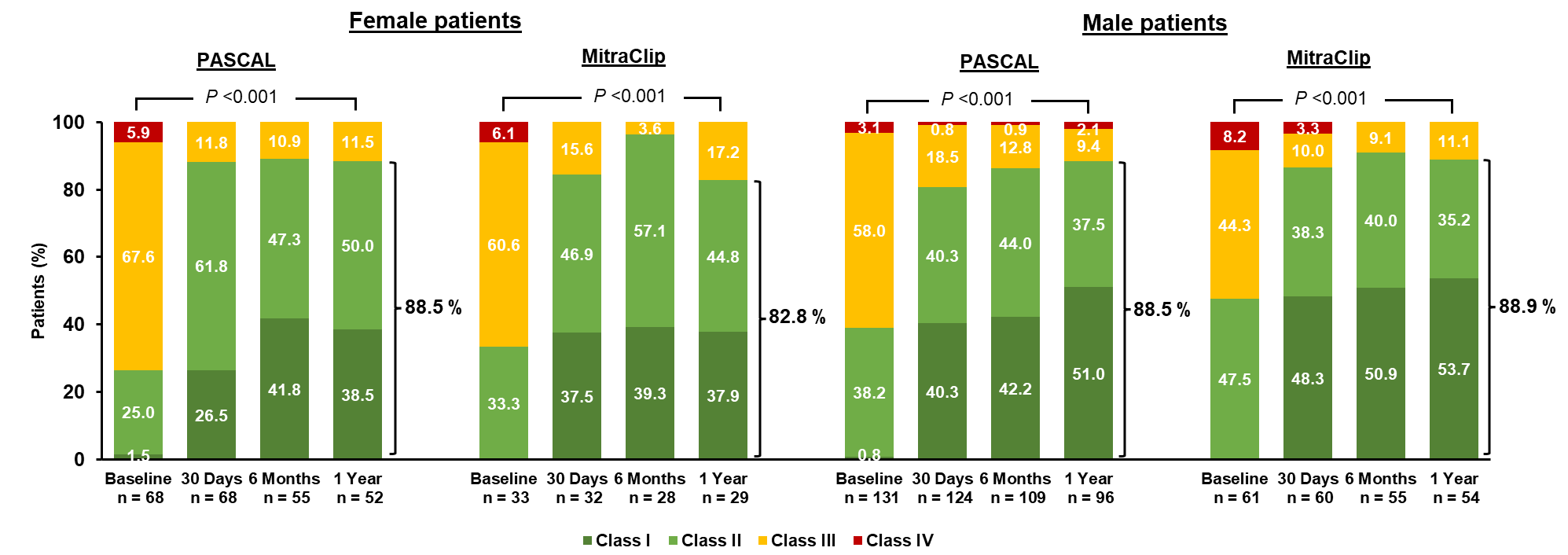


The graph shows unpaired analysis. *P* values for intragroup comparison were calculated from paired analysis using the Wilcoxon signed rank test. *NYHA*, New York Heart Association.

# **Supplemental Figure S7: KCCQ overall score at 1 year stratified by sex and treatment group.**


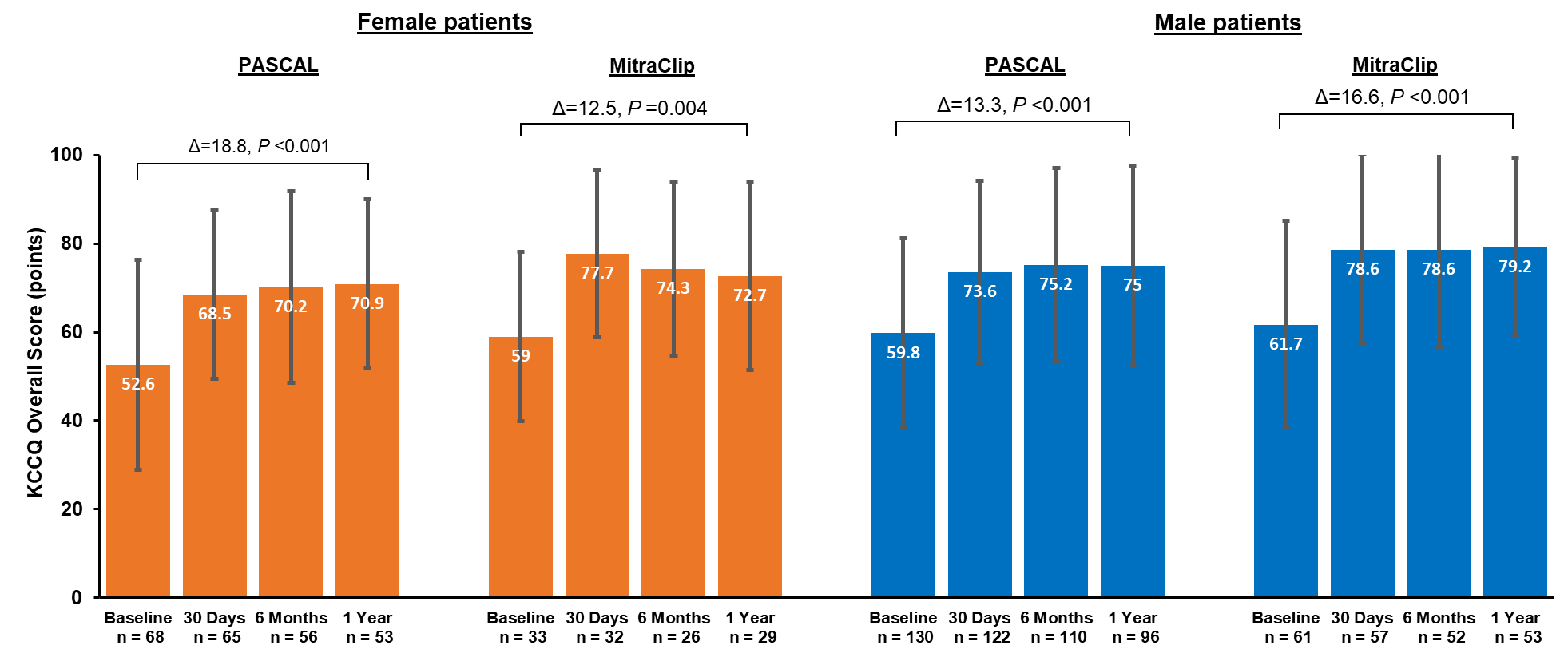


Graphs show unpaired analysis (mean ± SD). Δ denotes paired change from baseline, with intragroup *P* values calculated from Student’s t-test. *KCCQ*, Kansas City Cardiomyopathy Questionnaire.
